# Genome sequence of bacteriophage Janeemi isolated on *Arthrobacter globiformis* from soil collected in New York

**DOI:** 10.1128/mra.00177-24

**Published:** 2024-06-11

**Authors:** Yamini Patel, Vrushali P. Patel, Amna Syeda, Hannah Saji, Bryan P. Gibb

**Affiliations:** 1 Department of Biological and Chemical Sciences, New York Institute of Technology, Old Westbury, New York, USA; 2 Department of Psychology and Neuroscience, University of Colorado, Boulder, Colorado, USA; Portland State University, Portland, Oregon, USA

**Keywords:** bacteriophage, phage, SEA-PHAGE, actinobacteria, *Arthrobacter*, AZ, Janeemi, *globiformis*, *Arthrobacter globiformis*

## Abstract

Janeemi is a bacteriophage that infects *Arthrobacter globiformis* B-2880, which was isolated from soil collected in New York City. The genome has a length of 43,877 bp and contains 69 predicted genes. Based on gene content similarity to phages in the actinobacteriophage database, Janeemi is assigned to phage cluster AZ1.

## ANNOUNCEMENT

**Fig 1:**
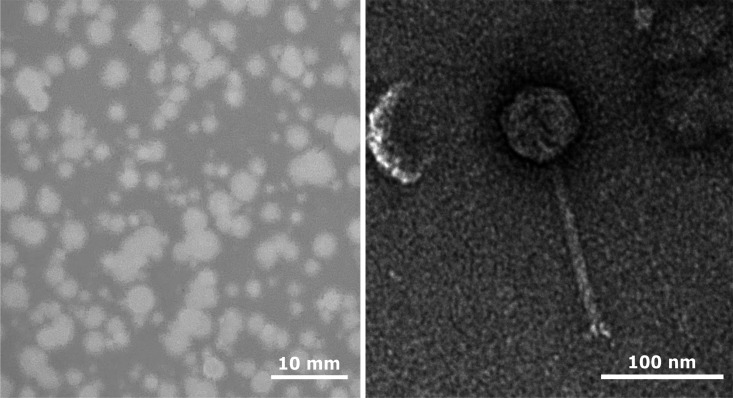
Plaque morphology (left) and transmission electron micrograph (right) of phage Janeemi. Phage plaques were imaged following incubation at 30°C for 48 h. Phage lysate was negatively stained with 1% uranyl acetate and imaged with a JEOL JEM-1400 transmission electron microscope at 120 KeV.

*Arthrobacter* phage Janeemi was isolated at the New York Institute of Technology in Old Westbury, NY, from soil sampled from around tree roots in Riverside Park, New York City, New York (40.801234 N, 73.97231 W), as part of the Science Education Alliance-Phage Hunters Advancing Genomics and Evolutionary Science (SEA-PHAGES) program (1). Phage isolation, plaque purification, and genome extraction were performed according to protocols in the SEA-PHAGES Discovery Guide (https://seaphagesphagediscoveryguide.helpdocsonline.com/home). The soil sample was washed in peptone-yeast-calcium (PYCa) liquid medium, the wash filtered (0.2-um pore size) and inoculated with *Arthrobacter globiformis* B-2880. After incubation with shaking at 30°C for 2 days, the culture was filtered, and the filtrate plated with *A. globiformis* B-2880 in PYCa top agar. Janeemi was purified by three sequential rounds of plating using a double-agar method with PYCa media. Following 2 days incubation at 30°C, Janeemi formed round (1–5 mm) turbid plaques (Fig. 1). High-titer lysates (>1 × 10^9^ pfu/mL) of Janeemi were produced from the original lysate by plate lysis on PYCa media. Janeemi can be stably stored at 4°C in phage buffer (10 mM Tris, pH 7.4, 10 mM MgSO_4_, 68 mM NaCl, 1 mM CaCl_2_) for more than a year with negligible loss in titer. Negative-staining transmission electron microscopy revealed a long (121.9 ± 6.9 nm; mean SD) flexible tail and (61.8 ± 1.0 nm; mean SD) icosahedral capsid (Fig. 1, *n* = 6) consistent with siphoviral morphology (2).

The genome for Janeemi was isolated from high-titer phage lysate using the Promega Wizard DNA cleanup kit prepared for sequencing with the NEBNext Ultra II FS Library Kit and sequenced using an Illumina MiSeq (v3 reagents) generating 244,917 150-bp raw unpaired reads. Reads were assembled using Newbler v2.9 (3) with default settings, generating a single contig of 43,877 bp with coverage of approximately 789. The contig was checked with Consed v29 (4) to evaluate the completeness and determine genomic termini. The genome of Janeemi is 67.10% GC and has defined 3’ single-stranded overhang ends of 5′-CGAACGGGCAT-3′. Based on shared gene content similarity (GCS) exceeding 35% from PhagesDB.org, Janeemi was placed into cluster AZ1 (5, 6). All bioinformatic analyses and software were used with default parameters. The coding regions were predicted using GeneMark v3.25 (7) and Glimmer v3.02b (8), and subsequently manually curated using DNA Master v5.23.6 (9), Phamerator (10), BLAST v2.7.1 (11), Starterator 1.2 (http://phages.wustl.edu/starterator/), and PECAAN v20211202 (https://blog.kbrinsgd.org/). No tRNA genes were identified with ARAGORN v1.2.41 (12). The functions for each coding sequence were predicted using NCBI BLASTP v2.9 (11), HHPred v3.2 (13), and Phamerator (10). Membrane proteins were predicted using TMHMM v 2.0 (14) and TOPCONS v2 (15).

Only 38 of the 68 predicted genes in Janeemi were assigned functions. All but one gene in Janeemi (39) are transcribed on one strand. Although most cluster AZ phages encode the endolysin in the middle of the genome, Janeemi is among a minority of cluster AZ phages (3/64 phages) to encode the endolysin at the beginning of the genome.

## Data Availability

Janeemi is available at GenBank with Accession No. ON970616 and Sequence Read Archive (SRA) No.SRX14483231.
